# Identification of Equid herpesvirus 2 in tissue-engineered equine tendon

**DOI:** 10.12688/wellcomeopenres.12176.2

**Published:** 2017-10-17

**Authors:** Roisin Wardle, Jane A. Pullman, Sam Haldenby, Lorenzo Ressel, Marion Pope, Peter D. Clegg, Alan Radford, James P. Stewart, Mohammed Al-Saadi, Philip Dyer, Mandy J. Peffers

**Affiliations:** 1 Institute of Veterinary Science, University of Liverpool, Leahurst Campus, Chester High Road, Neston, Wirral, UK; 2 Centre for Genomic Research, Institute of Integrative Biology, University of Liverpool, Biosciences Building, Crown Street, Liverpool, UK; 3 Institute of Ageing and Chronic Disease, University of Liverpool, William Henry Duncan Building, West Derby Street, Liverpool, UK; 4 Institute of Infection and Global Health, Department of Infection Biology, University of Liverpool, West Derby Street, Liverpool , UK

**Keywords:** tissue-engineered tendon, Equine herpesvirus 2, next-generation sequencing, superficial digital flexor tendon, equine

## Abstract

**Background:** Incidental findings of virus-like particles were identified following electron microscopy of tissue-engineered tendon constructs (TETC) derived from equine tenocytes. We set out to determine the nature of these particles, as there are few studies which identify virus in tendons *per se*, and their presence could have implications for tissue-engineering using allogenic grafts.

**Methods:** Virus particles were identified in electron microscopy of TETCs. Virion morphology was used to initially hypothesise the virus identity.  Next generation sequencing was implemented to identify the virus. A pan herpesvirus PCR was used to validate the RNASeq findings using an independent platform. Histological analysis and biochemical analysis was undertaken on the TETCs.

**Results: **Morphological features suggested the virus to be either a retrovirus or herpesvirus. Subsequent next generation sequencing mapped reads to Equid herpesvirus 2 (EHV2). Histological examination and biochemical testing for collagen content revealed no significant differences between virally affected TETCs and non-affected TETCs. An independent set of equine superficial digital flexor tendon tissue (n=10) examined using designed primers for specific EHV2 contigs identified at sequencing were negative. These data suggest that EHV is resident in some equine tendon.

**Conclusions:** EHV2 was demonstrated in equine tenocytes for the first time; likely from *in vivo* infection. The presence of EHV2 could have implications to both tissue-engineering and tendinopathy.

## Introduction

Tendons transfer force from muscle to bone, in addition to playing a key role in positioning and reducing locomotion expenditure, such as in the equine superficial digital flexor tendon (SDFT). Their structure is hierarchical ^1^, with the predominating component being a collagen-dominated extracellular matrix (ECM) ^2^. Sparsely distributed fibroblasts (tenocytes) produce ECM. Tenocytes are utilised in the production of tissue-engineered tendon constructs (TETCs), which are used within tendon research as a model for *in vivo* tendons, and have been explored as a potential therapeutic modality for tendinopathy ^3^.

There are few causes of musculoskeletal disease which have been attributed to viral infection, with no previous literature citing viral causes of tendinopathy in the horse. Previous viruses implicated in tendinopathy of other species include adenovirus and reovirus in tenosynovitis of broiler hens ^4^. This study in broiler hens suggests that a primary viral infection coupled with subsequent bacterial infection leads to catastrophic musculoskeletal infection and death.

Next generation sequencing (NGS) is frequently used as a research tool to characterise the transcriptome of eukaryotes, providing superior profiling to previously used methods such as microarrays. Wang *et al.* ^5^ describe NGS as a high throughput, highly sensitive method for transcriptome analysis. We have previously used it to characterise transcriptome-wide gene expression in numerous studies, including tendon ageing and disease ^6^. Additionally, it has infrequently been used to identify viral isolates within tissues ^7^ with a high sensitivity.

In this study equine TETCs were produced to report changing protein profiles with ageing. Following electron microscopy, two samples were found to contain virus-like structures. The aim of this study was to identify these particles using NGS and investigate their functional consequences on TETCs.

## Materials and methods

All reagents were supplied by Sigma unless otherwise stated.

### Sample collection

Equine SDFT was collected from young (n=7; age: 5 years ± 1.095 SD) and old (n=6; age: 18.5 years ± 2.429SD) donors from the equine hospital and abattoir as a by-product of the agricultural industry. The Animal (Scientific Procedures) Act 1986, Schedule 2 does not define abattoir collection as a scientific procedure and hence ethical approval was not required. Samples collected from an equine hospital were subject to the University of Liverpool ethical approval and consent (VREC462).

### Tissue-engineered tendon production

Tenocytes were digested from equine SDFT using standard collagenase protocol ^8^. Constructs were engineered as previously described by Kharaz *et al.* ^9^. In brief, tenocytes were seeded at 600,000 cells per well then scored every other day until full contraction of the construct. Constructs were harvested at 28 days and either snap frozen, collected for standard histology or transmission electron microscopy (TEM).

### Transmission electron microscopy

TEM of tendon constructs was performed following fixation in 2.5% glutaraldehyde in 0.1M sodium cacodylate buffer for 8 hours, followed by buffer washing procedures and second fixation and contrast stain with 0.1% osmium tetroxide for 90 minutes. Samples were stained with 8% uranyl acetate in 0.69% maleic acid for 90 minutes, dehydrated in ascending ethanol concentrations and embedded in epoxy resin. Ultrathin cross-sections (60–90 nm) were cut with a Reichert-Jung Ultracut on an ultramicrotome (Leica Microsystems, Wetzlar, Germany) using a diamond knife. Sections were then mounted on 200 mesh copper grids and stained with ‘Reynold’s Lead citrate’ stain for 4 minutes. Images were viewed in Philips EM208S Transmission Electron Microscope (Philips UK Limited, Guildford, UK) at 80K.

### Virion investigation

On examination of TEM images, two TETCs were identified as containing virus-like particles (V: Y1, Y6). These particles were morphologically assessed for virion identification. Particle diameter was estimated from electron micrograph images. Particle morphological characteristics were observed for virion diameter, presence of a viral envelope, nucleocapsid shape and surface projection presence. The number of full capsids, nucleocapsids and empty capsids were counted in 22–26 TEM images from each of the infected donors. For each sample, resin sections were mounted onto copper grids and viewed in the TEM at X 44000 magnification. 25–30 successive grid squares were viewed and the first area in each grid square which was found to contain virus was photographed. The images were loaded into ImageJ (version 1.51n) ^10^ and counts made of virus with:

i) envelope + nucleocapsid + DNA (full)

ii) envelope + nucleocapsid – DNA (nucleocapsid only),

iii) envelope only (empty).

TEM images were compared to current literature in order to provide a morphological reference ( Supplementary File 1).

### Histological analysis

TETCs were fixed in 4% paraformaldehyde and paraffin embedded. Sections were cut at 4µm onto polylysine slides and subsequently stained with haematoxylin and eosin (H&E) and Masson’s Trichrome. Histology was assessed using a scoring system developed in an unpublished report by Charters ^11^, and shown in Supplementary File 2.

### NGS analysis

Two samples of TETCs were submitted for RNASeq, one virally affected (V: Y1) and one control (NV). RNA was extracted from constructs as previously described, and NGS was performed in accordance with the method described by Peffers *et al.* ^6^. Analysis was undertaken by the Centre for Genomic Research, University of Liverpool. One µg of total RNA was ribosome depleted with the RiboZero Magnetic kit (Illumina, San Diego, California, United States). NGS libraries were prepared using the ScriptSeq v2 NGS Library Preparation Kit (Illumina, San Diego, California, United States). All of the enriched material was used as input material and following 15 cycles of amplification, libraries were purified using AMPure XP beads. Each library was quantified using Qubit and the size distribution assessed using the Agilent 2100 Bioanalyser (Agilent, Santa Clara, CA, USA). Libraries were pooled in equimolar amounts and quantity and quality of each pool assessed by using Bioanalyzer (Agilent, Santa Clara, CA, USA) and subsequently by qPCR using the Illumina Library Quantification Kit from Kapa (KK4854) on a Roche Light Cycler LC480II according to manufacturer’s instructions. The template DNA was loaded at 300 pM. The sequencing was carried out on one lane of an Illumina HiSeq4000 at 2×150 bp paired-end sequencing with v1 chemistry.

Initial processing and quality assessment was undertaken as previously described ^12^. Coverage plots were created using R. Initially a reference sequence file was created using known herpesvirus genomes. Reads were then aligned to the reference sequence files using Bowtie2 global alignment. PCR and optical duplicate reads were subsequently removed using Picard. Resulting alignment files were then used as input to Bedtools, (version 2.16.2) which was used to calculate coverage across the genomes. The coverage data output of this was used as input for a custom R script. Once the reads were mapped the number of reads mapped to each transcript was undertaken. These counts were calculated using HTSeq-count (version 0.6.1p1) ^13^, accepting only hits of quality 10+, and excluding ambiguous hits and hits on the opposite strand.

### NGS analysis: Transcript assembly and removal of host genomic reads

Illumina sequence reads were adapter- and quality-trimmed using Cutadapt version 1.2.1 (Martin, 2011) and Sickle version 1.200. Host sequences were removed by aligning trimmed reads to the Equus caballus genome (Equus caballus; EquCab2.56.pep) with HiSat (version 2.0.3b) ^14^.

### NGS analysis: Viral sequence determination

To taxonomically assign contigs, a BLASTN (MegaBlast, version 2.2.7+) ^15^) search of the assembled transcripts against the NT database from NCBI was carried out and full taxa information (species, order, phylum, superkingdom) was derived based on the best hit, using the NCBI taxonomy database ( ftp://ftp.ncbi.nlm.nih.gov/pub/taxonomy/taxdump.tar.gz). The search was carried out with an e-value cut off of 1e-5. The results were filtered to identify all hits to the virus superkingdom.

All raw read data produced in this study has been submitted to the EBI ENA, primary accession PRJEB20552, secondary accession ERP022713.

### Hydroxyproline assay

Freeze-dried samples (V (n=2) and NV (n=5) of TETC derived from the young donors were hydrolysed overnight at 60°C using papain reagent. Digests were stored at -20°C and subsequently assayed for collagen content by hydroxyproline assay ^16^.

### Statistical analysis

Data for the hydroxyproline assay were normality tested prior to statistical analysis. Data was considered statistically significant at *P*≤0.05. All normality testing and statistical analysis was undertaken using Excel (2010, Microsoft, Redmond, WA, USA) and GraphPad prism (2016, version 7, GraphPad Software Inc, CA, USA).

### Pan herpesvirus PCR

As RNAseq is not definitive, we used a pan-herpes PCR to confirm that the virus in question was indeed a herpes virus and there were no other herpesviruses present.

In order to validate the RNASeq findings with a different platform, a pan-herpesvirus PCR was undertaken. Samples V; Y1 and Y6 (virally affected) and a negative control (O3) were tested using a pan-herpesvirus PCR using a modified methodology described by Ehlers *et al.* ^17^. This protocol uses dI-substituted primers that offer improved sensitivity and specificity than previous protocols ^18^. **

Modifications of the components included using 12.5ul of 2x Qiagen multiplex PCR master mix containing HotStar Taq DNA polymerase (Qiagen, Crawley, UK). The list of primers used in pan herpesvirus PCR is available in Supplementary File 3.

### Specific EHV-2 PCR assay of equine SDFT

In order to determine the presence of EHV-2 in SDFT of a larger equine population, PCR primers were designed to amplify a region within a 37kb contig demonstrating an excellent depth of coverage (mean 4919x coverage) and 99% homology to EHV-2. The primer sequence was; V1 forward GGGCGGAGAATGTAGAGACG, V1 reverse GGTGGACTTTAACGGGGAGG (product size 443). DNA was extracted from 10 (mean ±SD age: 12.8±7.1) grossly normal equine SDFT collected from an abattoir. A QIAmp DNA extraction kit (Qiagen, Crawley, UK) following the manufacturer’s protocol was used. A sample of EHV-2 positive DNA (kindly gifted by Microbiology Diagnostic Laboratory, Institute of Veterinary Science, Liverpool) was used as a positive control.

PCR was performed in a final volume of 50ul consisting of 12.5ul ThermoPrime 2x ReddyMix PCR Master Mix, 1.5ul forward primer, 1.5ul reverse primer, 24.5ul water and 10ul extracted DNA template. Following an initial denaturation at 94°C for 9 min, products were amplified by 5 cycles of denaturation at 94°C for 1 min, annealing at 60°C for 1 min and elongation at 72°C for 1 minute. This was immediately followed by 30 cycles of denaturation at 94°C for 1 min, annealing at 55°C for one min and elongation at 72°C for one minute. Amplification was followed by a final extension at 72°C for 7 min. 15µl of product was electrophoresed on a 1.5% 1× Tris-acetate-EDTA agarose gel containing 10ul of PeqGreen. TrackIT 1Kb Plus DNA ladder was used as the standard.

### Phylogeny

All phylogenetic analysis was carried out within MEGA (version 5). DNA sequences were aligned using Clustal W (EMBL-EBI, Hinxton, UK). Neighbour-joining trees constructed. Bootstrap analysis (1,000 replicates) was used to provide support for individual nodes.

## Results

### Transmission electron microscopy

**Figure 1.**
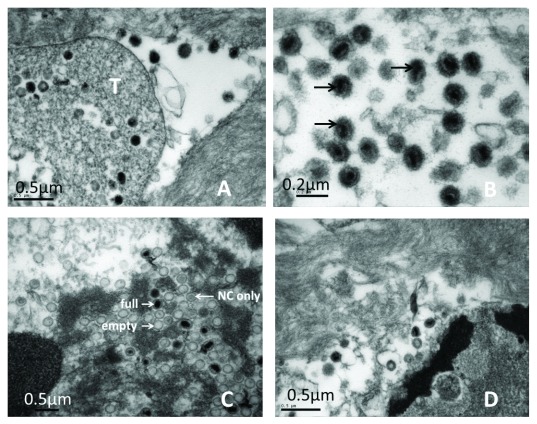
**A** and **B**: Electron micrographs of virus-like particles found within TETCs. Tenocyte (T), extracellular matrix viral particles (black arrows). **C** and **D**: Electron micrographs showing full capsids, nucleocapsids (NC) and empty capsids (white arrows) within both of the TETCs from which virus were isolated (C; Y1, D; Y6). Scale bars are shown.

Two out of 13 samples contained virus-like particles. The virus-like particles identified were approximately 100–150nm in diameter, and had an indistinct outer border with surface protrusions and a cylindrical nucleocapsid ( Figure 1A and 1B). Viral particles were identified both intracellularly (T) (including intranuclearly) and within the extracellular matrix ( Figure 1A). A further characteristic present in several of the images observed were ‘empty capsids’ within cells ( Figure 1C and 1D). Viral families have distinct morphological characteristics relating to size, nucleocapsid shape, and envelope present, which were used to tentatively identity the particles. The virus particles identified within the TETCs exhibited a spherical shape, with a visible envelope, contained within an icosahedral capsid shape. Virion diameter was calculated and was suggestive of a retrovirus or herpesvirus. The apparent spiked edge of the viral particles was suggestive of a retrovirus. In order to tentatively assess the level of infection within the TETCs, the number of tenocytes affected with virus particles was counted by examining the TEM images of all the donors. Whilst no virus particles were evident in images of the ten non-infected donors, 65.4% and 100% of the tenocytes of the two infected samples contained virus particles. The number of empty, full and nucleocapsids was determined for a set of images from both of the virus-infected samples. Results are shown in Table 1.

**Table 1.** Counting of full, empty and nucleocapsids in virus-infected TETC samples.

	Sample Y1			Sample Y6		
	Sum	Mean	SD	Sum	Mean	SD
**Full capsid**	164	7.5	4.5	316	12.2	8.3
**Nucleocapsid** **only**	129	5.9	5.1	183	7.0	5.3
**Full capsid +** **nucleocapsid**	293	13.3	5.5	499	19.2	10.6
**Empty capsid**	261	11.9	20.6	438	16.8	18.8
**Empty +** **nucleocapsid**	390	17.7	24.3	621	23.9	23.3

SD; standard deviation

### Histological scoring

**Figure 2.**
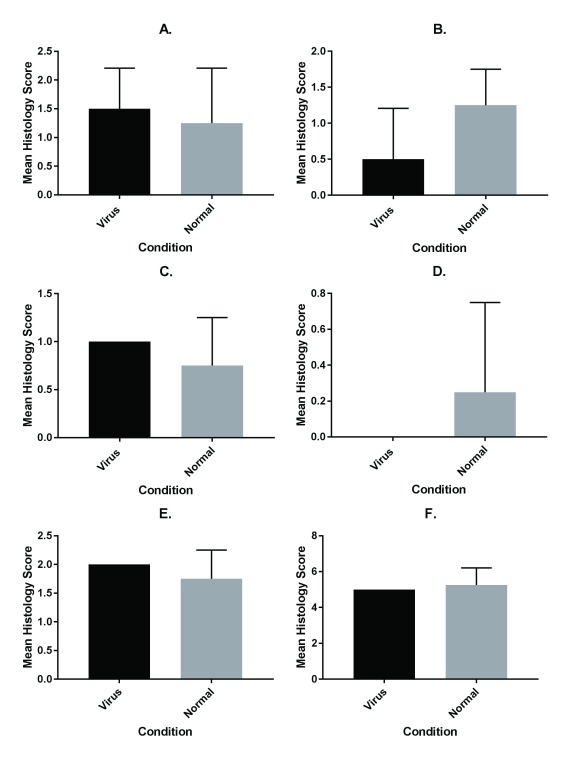
Histograms of histological scoring of TETCs. Histological scoring of TETCs from virally affected (n=2) and normal (n=8) donors. Graphs **A**– **F** represent the mean scores + standard deviation of the following characteristics; mean extracellular matrix organisation ( **A**), cell shape ( **B**), cellular distribution ( **C**), cellular alignment ( **D**), TETC cellularity ( **E**) and mean total score ( **F**). Where error bars are not present, scores for all donors were equal. Further details of the scoring system are available in Supplementary File 2. No significant differences were found between virally affected and normal donors (p≤0.05).

There were no significant differences in histological score between V and NV based upon the characteristics measured ( Figure 2).

### Hydroxyproline assay

**Figure 3.**
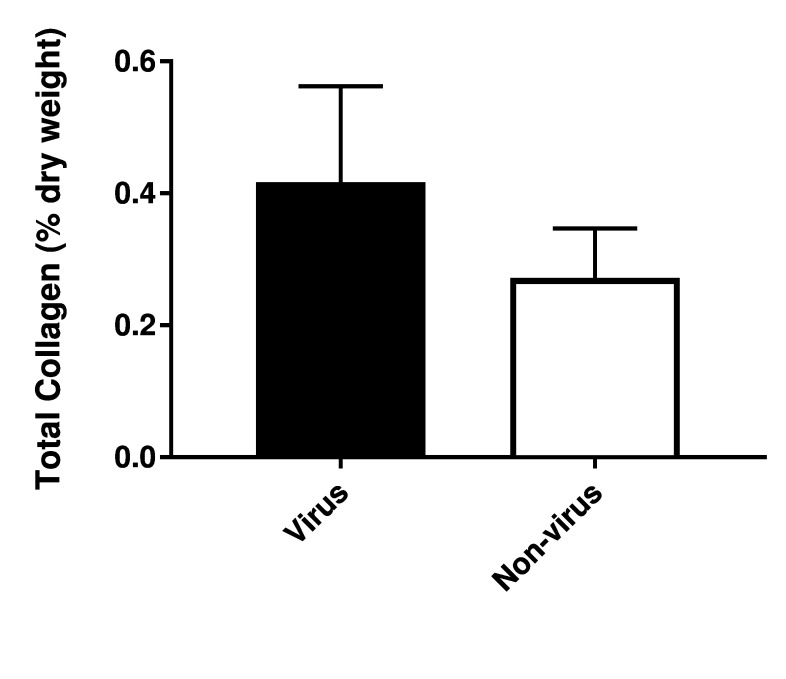
Histogram of collagen content of young TETCs containing virus (V; n=2) and not containing virus (NV; n=5). Graphs represent mean± standard error mean of percentage collagen normalised to dry weight. No significant difference was found in collagen content (p≤0.05).

Contrasting V and NV TETCs showed that there was no significant difference in collagen content ( Figure 3).

### NGS analysis

***NGS analysis: Transcript assembly and removal of host genomic reads.*** Illumina sequence reads were adapter- and quality-trimmed using Cutadapt version 1.2.1 (Martin, 2011) and Sickle version 1.200. A summary of raw and trimmed sequence data is shown in Supplementary File 4, following host sequence removal, and subsequent filtering of mapped reads. Resulting paired-end reads were assembled using Trinity version r2013_08_14 ^19^ for both samples. V resulted in 913,443 transcripts (including isoforms) and NV resulted in 788,076 transcripts (including isoforms).

***NGS analysis: Viral sequence determination.*** Y6 had 131 transcripts that hit viral sequences, 129 were assigned to Equid herpesvirus 2, a single read was assigned Equine Infectious Anaemia (EIA) virus and a single read to Bovine viral diarrhea virus (BVDV). For Y6 84.25% reads were mapped to the Equus caballus genome and 1.32% to Equid herpesvirus 2 strain G9/92 complete genome (accession; KM924294.1); the strain that most reads mapped to in this sample. Identified gene transcripts are in Table 2.

**Table 2.** The reads mapping to specific genes/transcripts in EHV2 (G9/92). Counts are presented in the table calculated using HTSeq-count.

Read Count	Gene Name	Product
0	E1	membrane protein E1
0	E10	apoptosis regulator E10
286	E2	protein E2
86	E3	membrane protein E3
867	E4	apoptosis regulator BALF1
34	E5A	protein E5A
3018	E6	membrane protein BILF1
0	E6A	protein E6A
853	E6C	protein E6C
260	E7	interleukin-10
1704	E7A	envelope glycoprotein 42
777	E8	apoptosis regulator E8
8	E9	membrane protein E9
4275	ORF10	protein G10
6163	ORF11	virion protein G11
5187	ORF17	capsid maturation protease
0	ORF17.5	capsid scaffold protein
2661	ORF18	protein UL79
6867	ORF19	DNA packaging tegument protein UL25
3165	ORF20	nuclear protein UL24
2484	ORF21	thymidine kinase
697	ORF22	envelope glycoprotein H
5154	ORF23	tegument protein UL88
13490	ORF24	protein UL87
3629	ORF25	major capsid protein
721	ORF26	capsid triplex subunit 2
736	ORF27	envelope glycoprotein 48
157	ORF28	envelope glycoprotein 150
21228	ORF29	DNA packaging terminase subunit 1
4507	ORF3	protein G3
9	ORF30	protein UL91
286	ORF31	protein UL92
971	ORF32	DNA packaging tegument protein UL17
1339	ORF33	tegument protein UL16
1194	ORF34	protein UL95
107	ORF35	tegument protein UL14
833	ORF36	tegument serine/threonine protein kinase
1325	ORF37	deoxyribonuclease
737	ORF38	myristylated tegument protein
2950	ORF39	envelope glycoprotein M
3897	ORF40	helicase-primase subunit
1333	ORF42	tegument protein UL7
2535	ORF43	capsid portal protein
5864	ORF44	helicase-primase helicase subunit
782	ORF45	tegument protein G45
492	ORF46	uracil-DNA glycosylase
26	ORF47	envelope glycoprotein L
1296	ORF48	tegument protein G48
861	ORF49	protein G49
5403	ORF50	protein Rta
538	ORF51	envelope glycoprotein 350
376	ORF52	virion protein G52
579	ORF53	envelope glycoprotein N
2793	ORF54	deoxyuridine triphosphatase
756	ORF55	tegument protein UL51
5083	ORF56	helicase-primase primase subunit
1693	ORF57	multifunctional expression regulator
935	ORF58	envelope protein UL43
1058	ORF59	DNA polymerase processivity subunit
3208	ORF6	single-stranded DNA-binding protein
990	ORF60	ribonucleotide reductase subunit 2
1362	ORF61	ribonucleotide reductase subunit 1
954	ORF62	capsid triplex subunit 1
4884	ORF63	tegument protein UL37
13692	ORF64	large tegument protein
1382	ORF65	small capsid protein
1095	ORF66	protein UL49
597	ORF67	nuclear egress membrane protein
292	ORF67A	DNA packaging protein UL33
1494	ORF68	DNA packaging protein UL32
1249	ORF69	nuclear egress lamina protein
1356	ORF7	DNA packaging terminase subunit 2
1328	ORF70	thymidylate synthase
3639	ORF73	nuclear antigen LANA-1
5	ORF74	membrane protein G74
3677	ORF75	tegument protein G75
1561	ORF8	envelope glycoprotein B
2966	ORF9	DNA polymerase catalytic subunit

***NGS analysis: Latency associated transcripts.*** Two different types of mapping were undertaken in an attempt to identify latency associated transcripts. Firstly a megablast run against the entire nt database to get taxonomic information, and secondly a blastx run against a database created of all know ERV genes in animals from NCBI (these sequences only appeared for human in NCBI). We assessed the two sequences in NCBI that result from a search for 'latency associated transcript and herpes' and ran a blastx for both samples against these. Neither resulted in any significant hits.

O3 had 7 transcripts aligned to virus sequences, all assigned Equid herpesvirus 2. A further BLASTN ^20^ search against all animal ERV sequences from the NCBI database was carried out to check that the contigs suggested to be herpesvirus were not ERVs. No significant hits to known ERVS were found for contigs assigned as EHV2 in the previous search.

**Figure 4.**
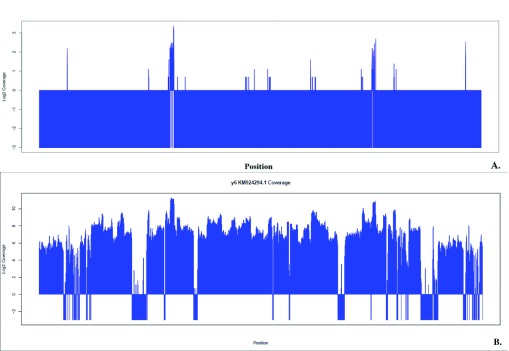
**A** and **B**. Log2-Coverage plot demonstrating read mapping. **A**. Read mapping plot of NV (O3). **B**. Read mapping plot of V (Y6). Both samples were mapped against the Equid herpesvirus 2 strain G9/92 (KM924294) genomes. Y-axes; coverage is log2-scaled. Zero coverage bases were assigned a log2-coverage value of -3 for plotting purposes.

***NGS: Genome coverage plots of discovered viral sequence against EHV2.*** Coverage plots of the samples were checked against the following genomes; Bovine herpesvirus type 1.1 (AJ004801.1,), Equid herpesvirus 1 strain T953 (KM593996.1), Equid herpesvirus 2 strain G9/92 (KM924294.1), Equid herpesvirus 3 strain AR/2007/C3A (NC_024771.1), Equid herpesvirus 4 (NC_001844.1), Equid herpesvirus 5 strain 2–141/67 (NC_026421.1). From these plots for samples NV (O3) ( Figure 4a) and V (Y6) ( Figure 4b), it is clear that the virus isolated from TETC Y6 is likely EHV2. There does not appear to be any EHV2 in the NV sample. Occasional coverage spikes were due to mapping low complexity reads which would map well against many genomes.

The coverage plot shows that the genomic coverage of the TETC virus of EHV2 strain G9/92 was 86.26%. The virus was mapped to 3,875.605 contigs to EHV-2, compared with 2–339 from the rest of the herpesvirus panel (EHV1, 3, 4, 5).

**Figure 5.**
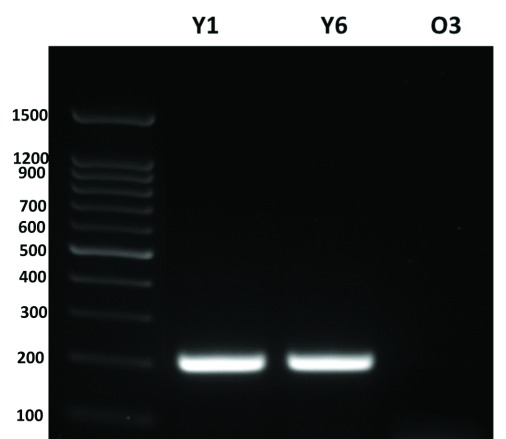
Pan herpesvirus gel image. Gel image of PCR fragments following restriction by ECOR 1. Virus-infected samples Y1, Y6 and virus negative sample O3 are shown. Amplicon size is 229 bp. Bands were removed and subsequently sequenced to confirm identification of herpes virus.

***Pan herpesvirus PCR.*** Pan herpesvirus PCR was found to be positive for EHV2 for the two V samples and negative for NV samples ( Figure 5). Sequencing of PCR products was used to confirm EHV2 presence within the sample.

**Figure 6.**
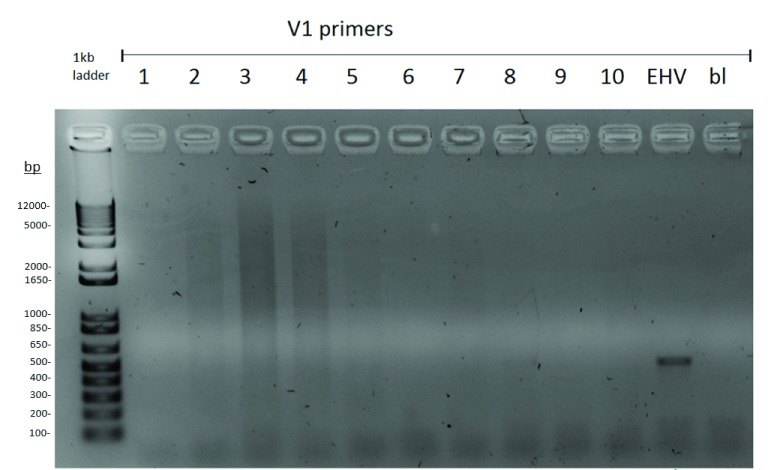
EHV2 PCR assay in an additional cohort of SDFT samples. DNA extractions from ten equine SDFT samples (1–10) were amplified with primers (V1) designed within a EHV-2 contig identified following NGS. Genomic DNA from EHV-2 was used as a PCR positive control (EHV) and water as a negative control (bl). TrackIT 1Kb Plus DNA ladder was used as a marker (1kb ladder). The positive EHV2 control demonstrates a band at 450bp.

***Specific PCR.*** Of the ten samples assessed, none were found to contain EHV2 ( Figure 6).

**Figure 7.**
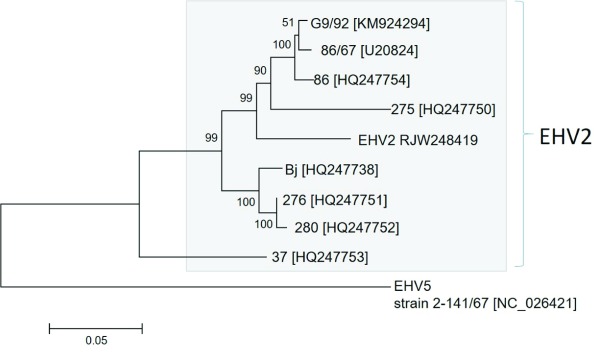
Neighbour-joining trees. Trees characterised the relationship between virus isolated from V (EHV2 RJW248419) and previously isolated strains using glycoprotein B gene. Bootstrap analysis (1,000 replicates) was used to provide support for individual nodes.

***Phylogenic analysis.*** A phylogenetic tree was produced in order to characterise the relationship between the TETC virus and currently identified EHVs ( Figure 7). Phylogenetic analysis of the TETC isolated virus glycoprotein B gene shows branching with Equid herpesvirus 2 strain 275, with further close relationships with strains G9/92, 86/87 and 86. The phylogenetic tree produced clearly demonstrates that the strain isolated from the TETCs is an EHV2, although not a strain which currently has been genome sequenced in the NCBI database.

## Discussion

This study is the first to identify EHV2 within cells derived from equine tendons. Findings of virus particles in samples of TETCs in which TEM was undertaken were incidental. At the time in our laboratory, equine tenocytes were the only cell type being cultured. We were interested in the nature of the particles and thus set out to investigate further. As this was a post-experiment attendant result identified a number of weeks after the end of the study, we were unable to undertake culture of the virus-infected tenocytes or determine what the titre of the virus was and if this was infectious. We realise this is a limitation of our work. However, as the presence of EHV2 was previously unreported in tendons, and due to the potential use of TETCs as allogenic transplant agents, we believe the finding of EHV2 in tenocytes could have implications for future tissue-engineering studies. Additionally, it should serve as a warning that viral contamination of musculoskeletal tissues may not be appreciated if screening is not undertaken.

Initial analysis tentatively identified a herpesvirus through electron microscopy, and this was confirmed as EHV2 with NGS and a pan-herpesvirus PCR. The structure of virion particles on TEM was initially suggestive of a retrovirus or a herpesvirus. Differential features based upon morphology included glycoprotein spikes, suggestive of a retrovirus ^21^, and undefined tegument protein, suggestive of a herpesvirus ^22^. We demonstrated the presence of empty capsid structures within both TETCs. Previous studies have hypothesised that empty capsids are precursors of mature herpesvirus ^23, 24^. Previous images identifying A-capsids ^24^ are similar to those in our study, further implicating identification of an equid herpes virus. This, coupled with measurement of virion diameter, was suggestive of a herpes or retrovirus ^25^. Due to the non-conclusive ultrastructural features in regard to differentiating between a herpesvirus and retrovirus, further analytical methods were employed to distinguish viral identity.

NGS identified the virus within the TETCs as most similar to EHV2 strain G9/92. Phylogenic analysis of the sequence isolated through NGS (EHV2 RJW 248419) demonstrated the close relationship between the virus in this study and the currently sequenced strains.

Swenson *et al.* ^26^ studied the presence of feline herpesvirus-1 (FHV1) within the feline tendon following experimental inoculation. It has previously been described that tissue-engineered tendons can be used as allographic transplant agents ^27^. They implied that the presence of FHV1 for application of TETCs in allogenic transplantation could lead to immune rejection of the engineered tissue and hence transplant failure. Whilst the histological structure and collagen content of the TETCs was not altered by the presence of EHV2, the ability of EHV2 within TETCs here to survive culture and multiple passages whilst retaining the ability to reproduce, may have implications for their use as a therapeutic option in terms of transplant rejection.

In the literature, isolation of primary virus within tenocytes is limited, though fibroblasts from other anatomical locations have demonstrated the ability for viral replication. Klevjer-Anderson *et al.* ^28^ describe the persistent infection of equine dermal fibroblasts with EIA. Further virus isolated from fibroblasts include herpes simplex virus within avian fibroblast cells. Stulberg *et al.* ^29^ discuss the growth of herpes simplex virus within cultured fibroblasts, illustrating a cytopathic effect upon cells cultured using a non-plasma technique. Such areas of focal necrosis were not evident in the TETCs here.

Viral causes of tenosynovitis have previously been described in chickens ^4^. The study investigated outbreaks of tenosynovitis in commercial broilers using virus isolation. Tendon from affected flocks yielded isolation of reoviruses and adenoviruses. To our knowledge, viral agents have not previously been isolated from tendons in horses. Zeng *et al.* ^30^ ** used TEM to visualise adenovirus particles. Their TEM images suggest that adenoviruses are much smaller than the particles isolated in this experiment, with the approximate diameter being 70–90nm. A similar result was evident with reoviruses’, which have an average diameter of 60–80mm.

Whilst all culturing was undertaken in sterile conditions the identified virions could have originated from a culture contaminant. Likely causes include cross-contamination between TETCs and other tissue within the laboratory or use of contaminated reagents during production. Previous studies have found viral contamination in commercially available foetal calf serum such as that used in TETC production here. However, as the virus was limited to a subset of cultured TETC this is unlikely. Viral isolates include BVDV, bovine parainfluenza virus-3 and bovine herpesvirus-1 (BoHV1) ^31, 32^. Membrane filtration removes many contaminants, however these may be an ineffective against viruses due to their small size ^33^.

It is important to consider host-range and tissue specificity of viruses, which could be present as a contaminant of tissue culture reagents. Foetal calf serum was used in the tissue-engineered culture system. As a result, we compared the viral reads obtained to a variety of bovine virus’ with suggestive morphology. One read to BoHV1 was identified in virally affected TETC. As an alpha-herpesvirus BoHV1 has previously shown limited scope for crossing species-barriers ^34^. The single read to BoHV1 when compared with EHV2 (129) suggest that BoHV-1 was not the virus isolated here. Moreover, the BoHV1 read could be a consequence of cross mapping. One read was found to EIA. This is a notifiable disease within the UK. The single read was of only 52bp hence it was suspected due to cross-mapping and further investigation was not pursued.

One additional potential explanation for the presence of EHV-2 in the samples is the ubiquitous nature of EHV-2 in both foals to adult horses ^35^. One might predict that it is not surprising to identify EHV-2 in tendon but perhaps more surprising that it was identified in only two of 13 samples. A number of studies have described equine gammaherpesviruses such as EHV-2 in peripheral blood mononuclear cells (PBMCs) ^36^. EHV-2 is latent in B lymphocytes and direct cell to cell contact between epithelial cells and B lymphocytes is required for the production of infection ^37^. In a study of Icelandic horses of EHV-2 in PBMCs and a selection of tissues using a co-cultivation technique 80% of horses examined demonstrated infectious replicating virus ^38^. These studies suggest that EHV-2 or cells containing the viral genes could have been present in a high proportion of tendon taken. Whilst every effort was made to remove tendon from the lower limbs during dissection in a aseptic manner cross contamination by other infected or latently-infected cells cannot be ruled out. Thus it is possible the tendon derived EHV-2 came from cell to cell contact of either PBMC or other donor cells and that this was the route of infection. However of the two infected samples (both from young donors) one was from the abattoir collection and one from the equine hospital. It would have been expected that more than one abattoir sample would be affected if this was the case as these were harvested, handled and processed at the same time.

Whilst the PCR for EHV-2 on tendon did not find any viral DNA this test cannot confirm lack of presence of latency associated transcript RNA. We did not identify the presence of latency associated transcripts in the RNAseq data. When the two sequences in NCBI that result from a search for 'latency associated transcript and herpes' were used neither resulted in any significant hits. Thus whilst latency associated transcripts cannot be ruled out with data currently available this is unlikely. However in order to confirm this a more in-depth analysis than this would require further research in latency associated transcripts linked to herpes in other animals which is beyond the scope of this study.

Unfortunately the EHV status of the donor horses was unknown. The clinical history for one of the TETC donors containing virus was available. The donor was presented at the hospital with right hind lameness and proprioceptive deficits. The horse was euthanased on humane grounds. EHV myeloencephalopathy was not suspected on presentation due to the absence of cauda equina signs ^39^. Moreover, the equid herpesvirus most associated with myeloencephalopathy is EHV1 ^40^. NGS data in this study revealed minimal coverage of the EHV1 genome when compared with EHV-2. There was no clinical history of the TETC donor as this was derived from an abattoir and hence further conclusions cannot be drawn. Since a small population of other horses were assessed for the presence of EHV2 RJW 248419 it would appear that the presence of this virus in equine tendon is uncommon.

Finally, an interesting point to consider is the potential role of an equid herpes virus in tendinopathy. EHV2 has not previously been isolated in cases of musculoskeletal disease in the horse. Whether this is because it has not been investigated or because it has no significant role in disease has not been established. Due to the unknown status of horse from the abattoir it is difficult to draw conclusions from the single case in which hind limb pathology was described. Whilst histological examination and limited biochemical tests within this study implies that EHV2 has no significant affect upon the structure of TETCs the mechanical properties of the TETCs were not assessed. Further work is required to determine if EHV-2 has a role in tendinopathy.

## Conclusions

This study identifies EHV2 in equine tendons for the first time, and describes NGS as a useful tool for virus identification. The implications of the presence of EHV2 in tendon to both tissue-engineering and tendinopathy requires further work. However, there are potential implications for the use of TETCs as allogenic transplant agents, as the presence of virus could result in transplant failure.

## Data availability

The data referenced by this article are under copyright with the following copyright statement: Copyright: © 2017 Wardle R et al.

All raw read data produced in this study has been submitted to the EBI ENA, primary accession PRJEB20552, secondary accession ERP022713. The data underlying this work has been uploaded to the Open Science Framework Database, and can be accessed via DOI, 10.17605/OSF.IO/WYPKQ ^41^.

## Supplementary material

Supplementary File 1. Table of the characteristics of various virus families that have previously been isolated in fibroblasts or as contaminants in tissue culture.

Supplementary File 2. Table showing the scoring system used during histological analysis of TETCs.

Supplementary File 3. Table of the primer sequences used in the pan herpesvirus PCR.

Supplementary File 4. Summary table of the raw and trimmed sequence data.
