# Supplementary material for: Identification of Equid herpesvirus 2 in tissue-engineered equine tendon
Source: Wellcome Open Res. 2017 Oct 17;2:60. Originally published 2017 Aug 3. [Version 2] doi: 10.12688/wellcomeopenres.12176.2 (PMC5664983; doi:10.12688/wellcomeopenres.12176.2)
Supplement: Supplementary file 1 [file wellcomeopenres-2-14023-s0000.tgz › 3b64c710-7c8e-42f2-a15c-2d9d041c8396.pdf]

Family Name	Nucleic acid	Naked/ Enveloped	Virion diameter (nm)	Capsid shape	Site of capsid assembly	Genome size (kb)
Reo	RNA	N	60-80	I	Cytoplasm	22-27
Flavi	RNA	E	40-50	I	Cytoplasm	10
Toga	RNA	E	60-70	I	Cytoplasm	12
Retro	RNA	E	80-130	I	Cytoplasm	3.5-9
Adeno	DNA	N	70-90	I	Nucleus	36-3
Herpes	DNA	E	120-200	I	Nucleus	120-200

Supplementary file 1. Characteristics of various virus families that have previously been isolated in fibroblasts or as contaminants in tissue culture (Flint *et al.*, 2015). I= Icosahedral
