# Supplementary material for: Identification of Equid herpesvirus 2 in tissue-engineered equine tendon
Source: Wellcome Open Res. 2017 Oct 17;2:60. Originally published 2017 Aug 3. [Version 2] doi: 10.12688/wellcomeopenres.12176.2 (PMC5664983; doi:10.12688/wellcomeopenres.12176.2)
Supplement: Supplementary file 2 [file wellcomeopenres-2-14023-s0001.tgz › 9013cfbf-e815-49a6-bd18-d33743295d96.pdf]

Characteristic	Score
Extracellular Matrix organisation<br>• Compact<br>• In part compact, in part loose<br>• Loosely composed, not orderly	 2<br>1<br>0
Cell Shape<br>• Spindle-shaped (normal)<br>• Mixture of spindle and round cells<br>• Oval to rounded shape	 2<br>1<br>0
Cell Distribution<br>• Homogenous Distribution of cells<br>• Focal areas of altered cell density	 1<br>0
Cellular Alignment<br>• Uniaxial<br>• More than 50% of cell with no uniaxial alignment	 1<br>0
Cellularity<br>• High Cellularity<br>• Intermediate Cellularity<br>• Low Cellularity	 2<br>1<br>0

Supplementary file 2. Scoring system used during histological analysis of TETCs.
