# Supplementary material for: Identification of Equid herpesvirus 2 in tissue-engineered equine tendon
Source: Wellcome Open Res. 2017 Oct 17;2:60. Originally published 2017 Aug 3. [Version 2] doi: 10.12688/wellcomeopenres.12176.2 (PMC5664983; doi:10.12688/wellcomeopenres.12176.2)
Supplement: Supplementary file 3 [file wellcomeopenres-2-14023-s0002.tgz › e85e3db5-ed13-486c-a156-1267fa62177a.pdf]

Primer	Type	PCR round	*Sequence 5’⟶ 3’
3-DFA	Modified	1	GAYTTYGCIAGYYTITAYCC
4-ILK	Modified	1	TCCTGGACAAGCAGCARIYSGCIMTIAA
8-KG1	Modified	1	GTCTTGCTCACCAGITCIACICCYTT
6-TGV	Modified	2	TGTAACTCGGTGTAYGGITTYACIGGIGT
10-IYG	Modified	2	CACAGAGTCCGTRTCICCRTAIAT
1-DFA	Unmodified	1	GAYTTYGCNAGYYTNTAYCC
2-ILK	Unmodified	1	TCCTGGACAAGCAGCARNYSGCNMTNAA
7-KG1	Unmodified	1	GTCTTGCTCACCAGNTCNACNCCYTT
5-TGV	Unmodified	2	TGTAACTCGGTGTAYGGNTTYACNGGNGT
9-IYG	Unmodified	2	CACAGAGTCCGTRTCNCCRTADAT

Supplementary file 3. Primers used in pan herpesvirus PCR
