# Supplementary material for: Identification of Equid herpesvirus 2 in tissue-engineered equine tendon
Source: Wellcome Open Res. 2017 Oct 17;2:60. Originally published 2017 Aug 3. [Version 2] doi: 10.12688/wellcomeopenres.12176.2 (PMC5664983; doi:10.12688/wellcomeopenres.12176.2)
Supplement: Supplementary file 4 [file wellcomeopenres-2-14023-s0003.tgz › 1fa76b6c-f79e-47ff-aec2-831ad5080933.pdf]

Sample ID	Untrimmed Reads	Trimmed Reads (% of untrimmed)	R1/R2 pairs	R0 reads (% of total trimmed reads)
Sample_1-y6	301,023,044	296,742,509 (98.58)	146,312,156	4,118,197 (1.39)
Sample_2-o3	344,778,884	338,216,303 (98.10)	166,053,650	6,109,003 (1.81)

Supplementary file 4. Summary of raw and trimmed sequence data.
