# Supplementary material for: Identification of Equid herpesvirus 2 in tissue-engineered equine tendon
Source: Wellcome Open Res. 2017 Oct 17;2:60. Originally published 2017 Aug 3. [Version 2] doi: 10.12688/wellcomeopenres.12176.2 (PMC5664983; doi:10.12688/wellcomeopenres.12176.2)
Supplement: Peer review history

# Peer review: Referee response for version 2

**DOI:** 10.21956/wellcomeopenres.14023.r27063

**Published:** 2017-10-27

**Authors:** Sarah Elizabeth Taylor

**Affiliations:** 1 The Royal (Dick) School of Veterinary Studies, University of Edinburgh, Edinburgh, UK

The authors have responded to comments from both reviewers and adjusted the manuscript accordingly such that a decision of approve can be made.

I have read this submission. I believe that I have an appropriate level of expertise to confirm that it is of an acceptable scientific standard.

# Peer review: Referee response for version 2

**DOI:** 10.21956/wellcomeopenres.14023.r27062

**Published:** 2017-10-25

**Authors:** Duncan Hannant

**Affiliations:** 1 School of Veterinary Medicine and Science, University of Nottingham, Loughborough, UK

The authors have taken attention of both reviewers’ comments. The paper status satisfies my recommendation of being accepted.

I have read this submission. I believe that I have an appropriate level of expertise to confirm that it is of an acceptable scientific standard.

# Peer review: Referee response for version 1

**DOI:** 10.21956/wellcomeopenres.13180.r26237

**Published:** 2017-10-09

**Authors:** Sarah Elizabeth Taylor

**Affiliations:** 1 The Royal (Dick) School of Veterinary Studies, University of Edinburgh, Edinburgh, UK

"This study suggests that a primary viral infection coupled with subsequent bacterial infection leads to catastrophic musculoskeletal infection and death" Please clarify that this is the study on broiler hens and not the current work.

 It is not clear from the current work what statistical tests were carried out in the programmes described in the materials and methods.

 Were any of the RNA seq hits for the latency associated transcript RNA?

 The specific PCR for EHV2 on tendon tissue did not find any viral DNA however can this test confirm lack of presence of latency associated transcript RNA?

 Was there any RNA from the virus infected tenocytes left over for analysis?

 Did the authors collect these tenocytes from tissue that was known to be septic antemortem? I wonder if this statement is a jump too far?

I have read this submission. I believe that I have an appropriate level of expertise to confirm that it is of an acceptable scientific standard, however I have significant reservations, as outlined above.

# Response

**Published:** 2017-10-13

**Authors:** Mandy Peffers

**Affiliations:** University of Liverpool

Reviewer

This study suggests that a primary viral infection coupled with subsequent bacterial infection leads to catastrophic musculoskeletal infection and death" Please clarify that this is the study on broiler hens and not the current work.

Response

We have amended the manuscript to clarify that this was indeed in broiler hens.

Reviewer

It is not clear from the current work what statistical tests were carried out in the programmes described in the materials and methods.

Response

We have amended the manuscript to clarify that these statistical tests relate to the hydroxyproline assay.

Reviewer

Was there any RNA from the virus infected tenocytes left over for analysis?

Response

All the RNA samples were used in the studies pertaining to this manuscript.

Reviewer

Did the authors collect these tenocytes from tissue that was known to be septic antemortem? I wonder if this statement is a jump too far.

Response

All samples were grossly normal on collection. Hospital derived samples showed no signs of sepsis.

Reviewer

Were any of the RNA seq hits for the latency associated transcript RNA?

Response

Two different types of mapping were undertaken. Firstly a megablast run against the entire nt database to get taxonomic information, and  secondly a blastx run against a database  created of all know ERV genes in animals from NCBI. Therefore the’ latency associated transcripts’ (only appear to be sequences for these in humans in NCBI) would not have been included. We assessed the two sequences in NCBI that result from a search for 'latency associated transcript and herpes' and ran a blastx for both samples against these. Neither resulted in any significant hits.  Thus whilst latency associated transcripts cannot be ruled out with data currently available this is unlikely. A more in-depth analysis than this would require further research in latency associated transcripts linked to herpes in other animals and is beyond the scope of this paper.  We have amended the manuscript accordingly.

Reviewer

The specific PCR for EHV-2 on tendon tissue did not find any viral DNA however can this test confirm lack of presence of latency associated transcript RNA?

Response

The reviewer is correct in their assumption. We have amended the manuscript.

# Peer review: Referee response for version 1

**DOI:** 10.21956/wellcomeopenres.13180.r24795

**Published:** 2017-08-29

**Authors:** Duncan Hannant

**Affiliations:** 1 School of Veterinary Medicine and Science, University of Nottingham, Loughborough, UK

This is a very well executed study carried out with high level of rigour to define and describe the findings of EHV-2 in equine tendon cells. There is one important aspect which potentially confounds the interpretation of the data. The authors refer to the possibilities of some contamination of samples and tissue culture reagents as a possible source of EHV-2 virus and PCR products in tendon cells and provide a good argument to discount these. However, one aspect is not fully considered in their justification of this potentially novel finding. There have been many published papers describing equine gammaherpes viruses in PBMC and several tissues/organs in both foals and adult horses. EHV-2 is ubiquitous in horses and virus and viral genes are commonly found. For example, Torfason et al. ^1^ described detection of EHV-2 and EHV-5 in PBMC and a selection of tissues in about 80% of the animals they examined using co-cultivation methods to demonstrate infectious replicating virus. EHV-2 is common in foals but these observations were also made in older horses on this occasion. As with other gammaherpes viruses, EHV-2 is known to be latent in B lymphocytes (Drummer et al. ^2^) and these latent viruses were re-activated and identified by co-cultivation methods. These and other findings strongly suggest that EHV-2 virus or cells containing EHV-2 genes would have been present in a high proportion of tissue samples taken from horses for this study. Many samples were from slaughterhouse materials but even so, it would have been very difficult to remove tendon tissues from horses without significant contamination by infected (or latently-infected) cells. 

 These possibilities should be discussed fully in the paper. It is possible also that cell-to-cell contact of infected PBMC or other donor cells with tenocytes is the route of infection of the latter in vivo. This would be perfectly acceptable as a method by which EHV-2 genes and virus were detected in cultured tenocytes. The fact that the authors detected virus in only 2 of the 13 samples might also be worthy of note, considering that this virus is so ubiquitous. 

  Do the authors know if the positive samples were from old or young horses?

 The considerations above do not detract from the findings that equine tendon cells may contain EHV-2 and that this might have implications for use of these cells therapeutically to treat tendinopathy. The contention is that the paper should discuss the findings in relation to published papers such as those indicated above and the potential likelihood of this finding not being unexpected.

I have read this submission. I believe that I have an appropriate level of expertise to confirm that it is of an acceptable scientific standard, however I have significant reservations, as outlined above.

## References

[1] TorfasonEGThorsteinsdóttirLTorsteinsdóttirSSvanssonV: Study of equid herpesviruses 2 and 5 in Iceland with a type-specific polymerase chain reaction. *Res Vet Sci*.2008;85(3) : 10.1016/j.rvsc.2008.01.003 605-11 10.1016/j.rvsc.2008.01.003 18336849

[2] DrummerHEReubelGHStuddertMJ: Equine gammaherpesvirus 2 (EHV2) is latent in B lymphocytes. *Arch Virol*.1996;141(3-4) :495-504 864509110.1007/BF01718313

# Response

**Published:** 2017-10-13

**Authors:** Mandy Peffers

**Affiliations:** University of Liverpool

The authors appreciate the comments made by the reviewer. We have taken on board their concerns and amended the manuscript accordingly to include discussion of other possible reasons for the findings of EHV-2 in equine tenocytes.
